# Somatic mutation counts as a surrogate marker for tumor mutation burden to predict progesterone receptor-positive (PR+) status in PIK3CA-mutated breast cancer

**DOI:** 10.3205/000352

**Published:** 2025-11-20

**Authors:** Bandana Kumari, Rijhul Lahariya

**Affiliations:** 1 Department of Biochemistry, All India Institute of Medical Sciences, Patna, Bihar, India; 2 All India Institute of Medical Sciences, Patna, Bihar, India

**Keywords:** breast invasive ductal carcinoma, PIK3CA mutation, tumor mutation burden, progesterone receptor, somatic mutation

## Abstract

**Objectives:**

Invasive ductal carcinoma (IDC), the most prevalent subtype of breast cancer, is characterized by significant genomic heterogeneity. Tumor mutation burden (TMB) has emerged as a predictive biomarker for immunotherapy response, yet its estimation via whole-exome sequencing remains complex and costly. This study aimed to evaluate whether total somatic mutation count can serve as a practical surrogate for TMB and assess its association with progesterone receptor (PR) status in PIK3CA-mutated IDC patients.

**Methods:**

This retrospective observational study utilized publicly available data from a previously published breast cancer sequencing study. A total of 164 female IDC patients with confirmed PIK3CA mutations and documented PR status were included. Relevant genomic and demographic parameters – TMB, mutation count, and age – were extracted and analyzed. Statistical analyses included correlation, intergroup comparisons by PR status, and binary logistic regression. Predictive performance was assessed using area under the receiver operating characteristic (AUROC) curves.

**Results:**

Patients with PR-negative status exhibited significantly higher TMB and mutation count than PR-positive patients (p-value<0.001 for both). TMB and mutation count were positively correlated (r=0.61, p-value<0.001), indicating overlapping representation of genomic instability. Logistic regression showed that mutation count was a significant predictor of PR status (p-value=0.01). Mutation count demonstrated a slightly superior predictive performance (AUROC=0.738) compared to TMB (AUROC=0.737).

**Conclusion:**

Total mutation count shows strong potential as a surrogate biomarker for TMB and a predictive marker for PR status in PIK3CA-mutated IDC, offering a cost-effective genomic tool in personalized breast cancer stratification.

## Introduction

Breast cancer is the most frequently diagnosed malignancy in women globally and a major contributor to cancer-related mortality [1]. Invasive ductal carcinoma (IDC), accounting for over 70% to 80% of all breast cancer cases, represents the most prevalent histological subtype [2]. Molecular classification based on immunohistochemical markers – estrogen receptor (ER), progesterone receptor (PR), and HER2 – divides breast cancers into distinct subtypes: luminal A, luminal B, HER2-enriched, and triple-negative [3]. These subtypes not only differ in prognosis and treatment response but also in their underlying genomic architecture [3].

The PIK3CA gene, located on chromosome 3q26.32, encodes the p110α catalytic subunit of phosphatidylinositol 3-kinase (PI3K), a key component of the PI3K/AKT/mTOR signaling axis [4]. Mutations in PIK3CA, particularly hotspot variants in exons 9 (helical domain, e.g., E545K) and 20 (kinase domain, e.g., H1047R), are present in up to 40% of ER+/PR+ tumours [5], [6]. These mutations promote oncogenic signaling through constitutive PI3K activation, contributing to hormone therapy resistance, enhanced proliferation, and altered metabolic states [7]. PIK3CA mutations often co-occur with other alterations, including MAPK pathway genes and CDH1, further modulating tumor phenotype and immune landscape [7].

Tumor mutation burden (TMB) – quantifying the number of somatic mutations per megabase of coding DNA – has emerged as a biomarker of genomic instability and neoantigen load [8]. However, TMB estimation requires large sequencing panels or whole-exome data, and is influenced by sequencing depth, coverage, and bioinformatic thresholds [9]. Conversely, total mutation count, the absolute number of somatic mutations per tumor, can be rapidly derived from variant call files with minimal preprocessing [10]. While both metrics reflect mutational load, their correlation and clinical interchangeability are not fully elucidated, especially in genetically stratified contexts like PIK3CA-mutated IDC.

Given the enrichment of PIK3CA mutations in hormone receptor-positive subtypes, it is biologically plausible that mutation burden may also associate with PR expression [11]. This study investigates whether mutation count correlates with TMB and if either metric can predict PR status. If mutation count demonstrates strong concordance with TMB and superior predictive accuracy, it may serve as a pragmatic, computationally accessible surrogate marker for hormone receptor profiling in breast cancer.

## Materials and methods

This was a retrospective observational study conducted using publicly available genomic and clinical data from the supplementary dataset of a previously published breast cancer sequencing study [12]. The dataset comprised somatic mutation profiles and hormone receptor status across a large cohort of breast cancer patients. From this resource, cases diagnosed specifically with invasive ductal carcinoma (IDC) and harbouring a confirmed PIK3CA mutation were selected for analysis. Only patients with clearly documented progesterone receptor (PR) status, assessed through immunohistochemistry (IHC), were included. After applying these criteria, a total of 164 female patients were identified and included in the final study cohort.

Relevant demographic and genomic parameters were extracted for each selected case. These included patient age, PIK3CA mutation status (positive/negative), total somatic mutation count, and tumor mutation burden (TMB), the latter reported in mutations per megabase as per the original study annotations. Cases with incomplete clinical records, missing PR status, or ambiguous mutation data were excluded from further analysis. Duplicate samples were also removed to ensure consistency and independence of data points. All data curation and pre-processing steps were performed.

### Statistical analysis

Data analysis was carried out using Jamovi version 2.6.44. The distribution of continuous variables was assessed using the Shapiro-Wilk test to determine normality. Depending on the distribution, continuous variables were summarized either as mean ± standard deviation (SD) or as median with interquartile range (IQR). Categorical variables were described using frequencies and percentages. Patients were divided into two groups based on PR status (positive or negative). Comparisons between the groups were made using the Independent t-test for normally distributed continuous variables or the Mann-Whitney U test for non-normally distributed data. For categorical variables, the Chi-square test was used. The relationship between continuous variables, including mutation count and TMB, was analyzed using either Pearson’s correlation coefficient or Spearman’s rank correlation, depending on the data distribution. A p-value ≤0.05 was considered statistically significant. To evaluate the ability of TMB and mutation count to predict PR status, binary logistic regression analysis was performed. Results were expressed as odds ratios (ORs) with 95% confidence intervals (CIs). The discriminative performance of each model was assessed using the area under the receiver operating characteristic (AUROC) curve, and performance metrics like accuracy, sensitivity and specificity were evaluated for comparison.

## Results

A total of 164 female patients with IDC and confirmed PIK3CA mutations were included in the final analysis. The cohort was stratified based on progesterone receptor (PR) status, with 122 patients (81.9%) being PR-positive and 27 patients (18.1%) PR-negative. The mean age of patients was 58.4±13.9 years, with no statistically significant difference between the PR-positive and PR-negative groups (p-value=0.672). The median TMB was 1.15 (IQR: 0.8–2.18) mutations/Mb, and the median total mutation count was 34 (IQR: 24–65.3). PR-positive patients tended to have lower TMB and mutation counts compared to PR-negative patients; and the difference was statistically significant for mutation count and for TMB (**p-value<0.001**) (Table 1 (Tab. 1)).

We evaluated age as a potential confounding variable in the association between genomic features and PR status. However, no significant relationship was found between patient age and PR positivity or negativity, indicating that age did not pose a confounding risk in this dataset and could be excluded from further adjusted analyses. We applied Spearman rank correlation test between TMB and mutation count. We found a strong positive correlation between TMB and total mutation count (r=0.998, **p-value<0.001**), reinforcing the biological plausibility that both metrics capture aspects of tumor genomic instability. This relationship suggests that mutation count, despite being simpler to calculate and more widely available across datasets, may serve as a reliable proxy for TMB, particularly in resource-limited or retrospective genomic studies.

Binary logistic regression analysis showed that both mutation count and TMB models significantly predicted PR status in PIK3CA-mutated IDC patients, with the overall model fit reaching statistical significance (χ^2^=6.71, **p=0.01**) for both models. This indicates that incorporating either mutation count or TMB into the predictive model improved the ability to classify patients according to their PR status compared to a null model without predictors.

**Figure 1:**
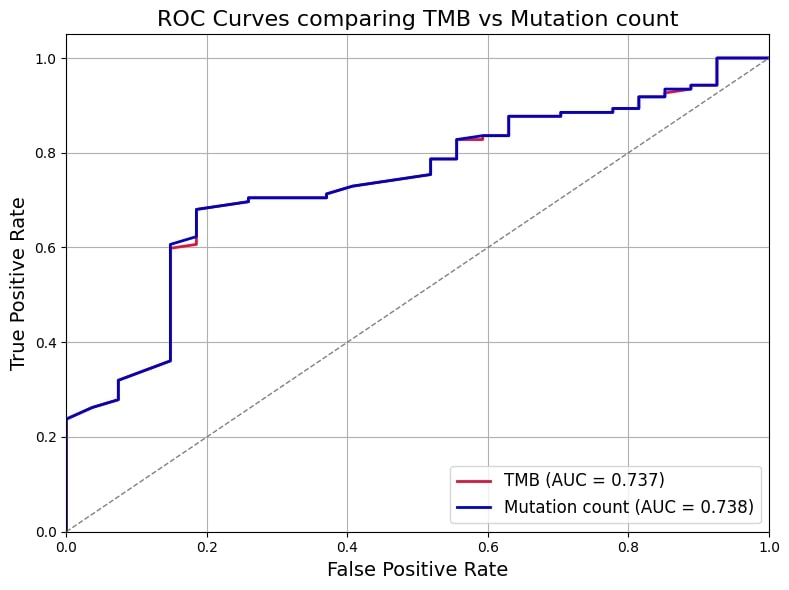
Comparison of ROC curves among TMB and mutation count

When comparing the two predictors, mutation count demonstrated a slightly better performance than TMB in discriminating PR-positive from PR-negative cases. This was reflected by a marginally higher AUROC of 0.738 for mutation count, compared to 0.737 for TMB. Although the difference is small, the higher AUC indicates a better overall ability of mutation count to correctly classify patients’ PR status. Additionally, both models showed comparable classification metrics, with an accuracy of 71.1%, sensitivity around 70.5%, and specificity approximately 74.1%, further supporting their similar predictive capacities (Table 2 (Tab. 2)). Therefore, the findings suggest that mutation count could serve as a practical and reliable surrogate marker for PR status prediction in PIK3CA-mutated breast cancer, potentially facilitating more accessible molecular profiling in routine clinical practice without compromising predictive accuracy. Figure 1 (Fig. 1) shows a comparison of ROC curves among TMB and mutation count. 

## Discussion

IDC is the most common histological subtype of breast cancer and displays considerable molecular heterogeneity [12]. Among its defining molecular alterations, PIK3CA mutations are frequently observed and are known to activate the PI3K/AKT/mTOR pathway, promoting tumor growth, survival, and endocrine resistance [7]. In our study focusing exclusively on PIK3CA-mutated IDC cases, we aimed to evaluate whether total somatic mutation count could serve as a surrogate for TMB in predicting PR status. We observed a significant positive correlation between TMB and mutation count, indicating shared reflection of genomic instability. Although both TMB and mutation count were statistically significant in predicting PR status when assessed independently, mutation count demonstrated slightly superior discrimination (AUROC: 0.738 vs. 0.737), supporting its use as a potentially simpler and more accessible predictor of hormone receptor profile in precision oncology.

PIK3CA is among the most frequently mutated genes in hormone receptor-positive breast cancers, including IDC [7]. Mutations in PIK3CA – especially those in helical (E542K, E545K) and kinase domains (H1047R) – are known to drive oncogenic transformation and have implications for targeted therapy, particularly with PI3K inhibitors such as alpelisib [6]. These mutations also affect the interplay between estrogen receptor signaling and PI3K pathway activation, thereby influencing PR expression and hormone responsiveness [13]. Notably, PR negativity in breast cancer has been associated with more aggressive behavior, reduced endocrine therapy response, and worse prognosis [14]. Thus, understanding the genomic correlates of PR status, particularly in PIK3CA-mutated contexts, has therapeutic relevance.

TMB has emerged as a pivotal biomarker in oncology, particularly for predicting responses to immune checkpoint inhibitors (ICIs) [15]. High TMB is often associated with an increased neoantigen load, enhancing tumor immunogenicity and potentially leading to better responses to immunotherapy [15]. However, the clinical utility of TMB is nuanced and varies across cancer types [16]. For instance, while high TMB correlates with improved outcomes in cancers like melanoma and non-small cell lung cancer, its predictive value in breast cancer remains less clear [15]. Assessing TMB typically requires comprehensive genomic profiling, such as whole-exome sequencing, which can be cost-prohibitive and technically demanding [17]. This has led to interest in alternative metrics like total mutation count, which, while less granular, can be more readily obtained from targeted sequencing panels. Studies suggest that total mutation count may serve as a practical surrogate for TMB, capturing aspects of genomic instability and offering predictive insights, especially in settings where comprehensive sequencing is not feasible [18].

In the context of breast cancer, particularly IDC, PIK3CA mutations are among the most common genetic alterations [11], [19]. These mutations activate the PI3K/AKT/mTOR pathway, contributing to tumor growth and survival [20], [21], [22]. While PIK3CA mutations have been associated with hormone receptor-positive subtypes, their relationship with TMB and mutation count in predicting treatment response warrants further investigation [23], [24]. Understanding the interplay between these genetic factors could enhance prognostic assessments and inform therapeutic strategies in IDC.

This study offers a novel perspective by demonstrating that total mutation count, a more accessible and cost-effective metric than traditional TMB, may serve as a practical surrogate for genomic instability in IDC. Unlike TMB, which requires whole-exome sequencing, mutation count can be derived from routine sequencing platforms, making it feasible for broader clinical use. By highlighting its comparable predictive performance for PR status, our findings support the integration of mutation count into diagnostic workflows, potentially enhancing patient stratification and guiding personalized therapeutic strategies in resource-limited settings. Clinically, this study supports using mutation count as a simpler, cost-effective surrogate for TMB to assess genomic instability. It may aid in hormone receptor-based risk stratification and treatment planning in invasive ductal carcinoma.

## Conclusion

This study highlights the potential of total mutation count as a clinically relevant surrogate biomarker for TMB in IDC patients harbouring PIK3CA mutations. While both TMB and mutation count showed significant association with progesterone receptor (PR) status, mutation count demonstrated a marginally superior predictive performance based on AUROC analysis. Given the logistical and financial constraints of calculating TMB through whole-exome sequencing, mutation count offers a practical, accessible alternative using targeted sequencing data. This approach may help streamline genomic profiling in resource-limited settings and enhance the identification of hormone receptor-related molecular subtypes. Importantly, our findings underscore the value of simple genomic parameters in guiding clinical decisions and refining personalized therapeutic strategies. By proposing mutation count as a surrogate marker, this study opens new avenues for integrating genomic data into routine breast cancer care, particularly for molecular stratification and potential immunotherapeutic implications in hormone-responsive breast tumors.

## Notes

### Authors’ ORCIDs

- Dr. Bandana Kumari: 0000-0001-5395-413X
- Dr. Rijhul Lahariya: 0009-0003-5769-4509

### Authors’ contributions

Bandana Kumari contributed to the study conception, methodology and draft preparation. Bandana Kumari and Rijhul Lahariya contributed in data collection. Data analysis was performed by Rijhul Lahariya. The first draft of the manuscript was written by Bandana Kumari and Rijhul Lahariya. Both authors read and approved the final manuscript. Rijhul Lahariya and Bandana Kumari have contributed equally to this work.

### Availability of data and materials

The datasets used and analysed during the current study are available from the corresponding author on reasonable request.

### Competing interests

The authors declare that they have no competing interests.

## Figures and Tables

**Table 1:**
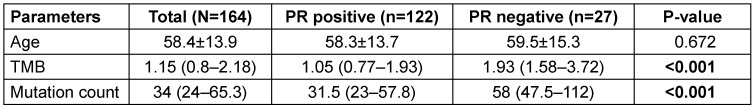
Baseline characteristics and comparison of age, TMB, and mutation count between PR-positive and PR-negative groups in PIK3CA-mutated breast IDC patients

**Table 2:**
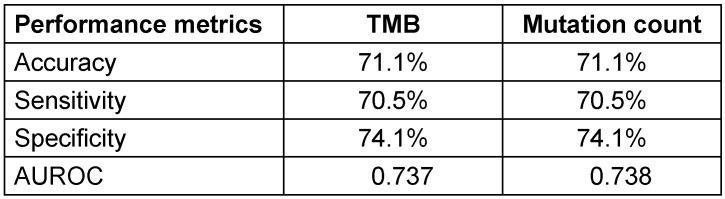
Comparison of predictive performance metrics for TMB and mutation count in classifying PR status among PIK3CA-mutated breast IDC patients
